# Giant Cell Tumor in the Thoracolumbar Junction: A Case Report

**DOI:** 10.7759/cureus.98546

**Published:** 2025-12-05

**Authors:** Victor M Villalba-Piña, Javier Gomez-Farias, Roxana G Figueroa-Baca, Felipe Aguilar-Chávez

**Affiliations:** 1 Department of Orthopedics and Traumatology, Hospital General de Ciudad Juarez, Ciudad Juarez, MEX; 2 Department of Neurology, University of Texas Health Science Center at Houston, Houston, USA; 3 Department of Orthopedics and Traumatology, Cisne Spine Academy, Star Medical Hospital, Autonomous University of Chihuahua, Chihuahua, MEX

**Keywords:** giant cell tumor, kyphosis, osteotomy, paraplegia, spinal neoplasms

## Abstract

A giant cell tumor (GCT) of the spine is an uncommon benign bone neoplasm that can behave aggressively and cause neurological compromise. Its rarity and location within load-bearing, neuro-critical anatomy make diagnosis and management particularly complex. A 34-year-old woman presented with progressive thoracolumbar pain, kyphotic deformity, and incomplete spinal cord injury leading to transient paraplegia. Radiographs showed 90% collapse of the L1 vertebral body with about 45° regional kyphosis. Magnetic resonance imaging revealed an expansile lytic lesion at L1 with paravertebral extension and severe canal compromise.

Definitive management was achieved through a posterior-only approach using a Schwab type five osteotomy, mesh-cage reconstruction with autograft, and T12-L4 instrumentation. Postoperatively, the patient showed rapid sensory recovery below T11 and preserved motor function, regaining ambulation within 24 hours. Follow-up imaging confirmed correction of deformity and construct stability. Histopathology demonstrated multinucleated giant cells in a mononuclear stromal background, confirming GCT. Locoregional recurrence was identified eight months postoperatively. This case highlights the value of prompt recognition and meticulous surgical planning in spinal GCT. Early intervention with complete resection and stabilization can restore alignment, prevent permanent neurological deficits, and achieve durable local control. Multidisciplinary management is crucial for optimizing outcomes in these rare but challenging lesions.

## Introduction

Spinal tumors are rare but clinically significant neoplasms that, without treatment, can cause severe neurological morbidity. They are classified based on their relationship to the spinal cord and dura mater as extradural (the most common at 50%), intradural-extramedullary, and intramedullary. The vast majority are metastatic (up to 97% of all vertebral lesions), with those originating from the breast, lung, and prostate being the most frequent [1-4].

In contrast, primary spinal tumors are rare and may arise from the spinal cord, meninges, or bony and soft-tissue structures. Malignant primary neoplasms include chordoma, chondrosarcoma, Ewing sarcoma, and osteosarcoma, while benign lesions include osteoid osteoma, osteoblastoma, aneurysmal bone cyst, osteochondroma, and giant cell tumor (GCT) [5].

When vertebral bodies are involved, lesions may produce vertebral destruction syndrome (VDS), structural instability, deformity, and neurologic compromise from infectious, metabolic, or neoplastic causes. Clinical evaluation should be paired with prognostic scores (Karnofsky, Tomita, Tokuhashi) to gauge function, metastatic burden, and survival, guiding treatment planning [5,6].

According to the World Health Organization (WHO), GCT is a Grade II benign but locally aggressive bone neoplasm composed of mononuclear stromal cells, multinucleated giant cells resembling osteoclasts, and spindle-shaped stromal cells. Its biological behavior complicates management because of its propensity for local recurrence and, rarely, metastasis. GCT accounts for ~5-6% of primary bone tumors; ~7-15% of GCTs arise in the spine, typically in the second to fourth decade with a slight female predominance [7-9].

Spinal GCTs, particularly those of the thoracolumbar region, present unique diagnostic and therapeutic challenges. The anatomical complexity of this junction, together with the proximity to neural and vascular structures, makes complete resection difficult and increases the risk of recurrence. The literature reflects uncertainty and a lack of consensus regarding the most appropriate diagnostic strategies, surgical approaches, and adjuvant therapies for these tumors [8,9]. The management of a GCT in the spine involves multidisciplinary participation by physicians such as spine surgeons, oncologists, and surgical oncologists. The first step is to perform a biopsy to confirm the diagnosis, with evidence supporting percutaneous biopsy as the first option. Once the diagnosis is confirmed, a scheme involving the use of denosumab for three to six months as an adjunct, combined with wide margin resection, is warranted [10,11].

The aim of this study is to delineate an evidence-based diagnostic pathway for GCT of the spine, emphasizing critical differential diagnoses and key decision points. By outlining the appropriate sequence of diagnostic evaluation, we aim to equip clinicians with the necessary conceptual and practical framework to guide accurate early diagnosis and optimal initial management.

## Case presentation

We present the case of a 34-year-old woman from Oaxaca, Mexico, a tuberculosis-endemic region. Her medical history included hyperthyroidism under treatment with thiamazole, with no substance use, complete vaccination status, and no other significant comorbidities. She reported one year of intermittent thoracolumbar pain and progressive kyphotic deformity, with marked worsening over the last four months. Eight months prior, she experienced a transient episode of paraplegia with total recovery. 

At presentation, she described moderate to severe midline axial pain and a visual analogue scale (VAS) score of six to eight out of 10 at the T12-L2 level radiating to both lower limbs, more pronounced on the left side. On neurological examination, muscle strength was three out of five on the Medical Research Council Scale (MRC) from L2 distally in both lower limbs, and sensory function was reduced (one out of two) from L1 downward bilaterally. The Oswestry Disability Index (ODI) score was 44%, and the Roland-Morris Disability Questionnaire score was 19/24. She denied weight loss, night sweats, or other systemic symptoms.

Preoperative laboratory tests, including coagulation profile, hematology, renal and hepatic function, and viral serologies, were within normal limits. Prognostic evaluation showed a Karnofsky Performance Status of 80%, Tokuhashi score of nine, Tomita score of four, and Skeletal Oncology Research Group (SORG) survival index predicting 99% at 30 days, 98% at 90 days, and 88% at one year.

**Figure 1:**
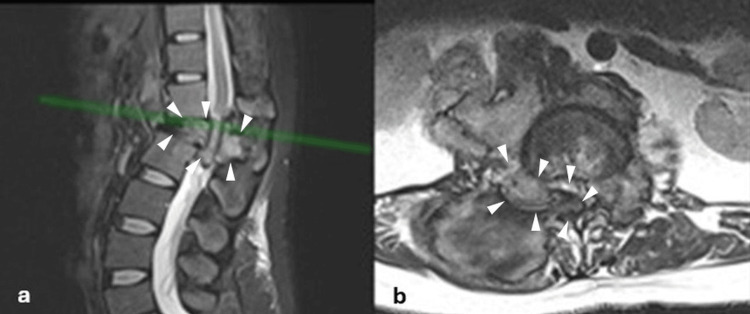
Sagittal and axial magnetic resonance imaging (MRI) of the lumbar spine at the initial encounter a) Sagittal T2-weighted MRI showing a space-occupying vertebral body lesion at the L1 level (arrowheads) with vertebral body collapse and thecal sac compression, with a 45° regional kyphosis. The green reference line indicates the axial slice level displayed in panel b; b) Corresponding axial T2-weighted MRI at the same level demonstrates the intracanal lesion (arrowheads), resulting in marked narrowing of the spinal canal and compression of the adjacent neural structures.

**Figure 2:**
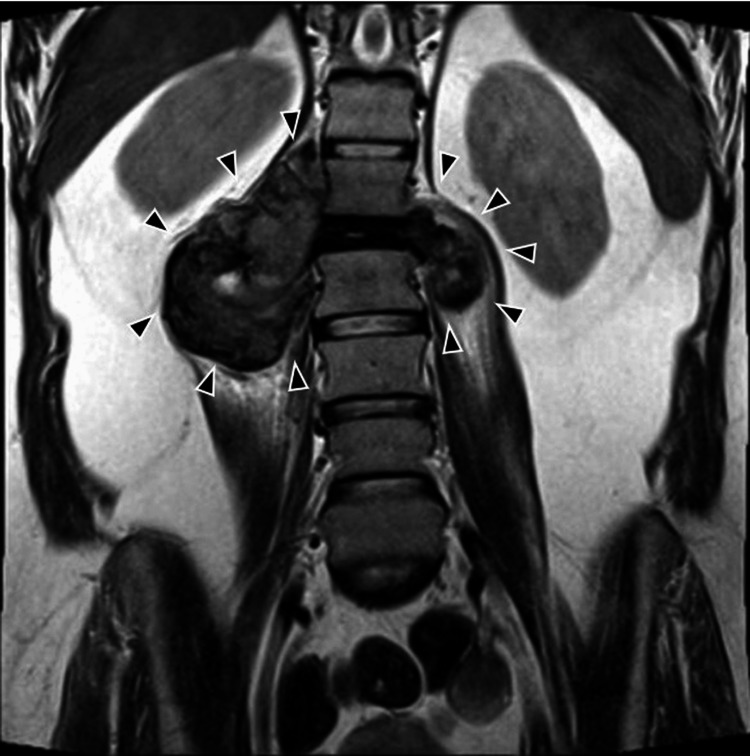
Preoperative coronal MRI of the lumbar spine, demonstrating a space-occupying lesion MRI, coronal view, T2-weighted sequence showing bilateral invasive expansion at the T1 vertebral body level, with a right-dominant mass effect and invasion of the iliopsoas muscle. The arrowheads delineate the lesion.

Initial X-rays revealed 90% collapse of the L1 vertebral body with regional kyphosis of 45°. MRI demonstrated an aggressive lesion involving the L1 vertebral body with collapse, canal invasion, and surrounding soft-tissue extension. On T2-weighted images, the lesion was predominantly hypointense with heterogeneous signal (Figures 1a, 1b, 2). CT with 3D reconstruction confirmed an osteolytic L1 lesion.

Two days after evaluation, an initial percutaneous biopsy suggested tuberculosis due to Ziehl-Neelsen positivity with granulomatous features. However, due to progressive neurological compromise and structural instability, definitive surgery was performed one week later (May 6, 2024).

Through a posterior approach, we performed an L1 corpectomy (Schwab type five osteotomy) with a mesh cage and graft placement, followed by T12-L4 instrumented fusion. A posterior approach and instrumentation were preferred because the surgical plan was to perform a long-segment instrumentation. This approach was sufficient to correct the kyphosis and maintain sagittal balance. The posterior approach allowed for a posterior corpectomy or a circumferential resection (often en bloc or marginal), which is the gold standard for minimizing recurrence, especially in the thoracolumbar spine. 

Intraoperative findings included a 50-gram right-dominant paravertebral tumor with similar tissue in the epidural space. Operative time was 4.5 hours with an estimated blood loss of 1,800 mL. The staining and culture were repeated with the definitive sample taken during the surgical instrumentation, which resulted in a negative histopathology for *Mycobacterium tuberculosis *and a positive diagnosis for GCT.

**Figure 3:**
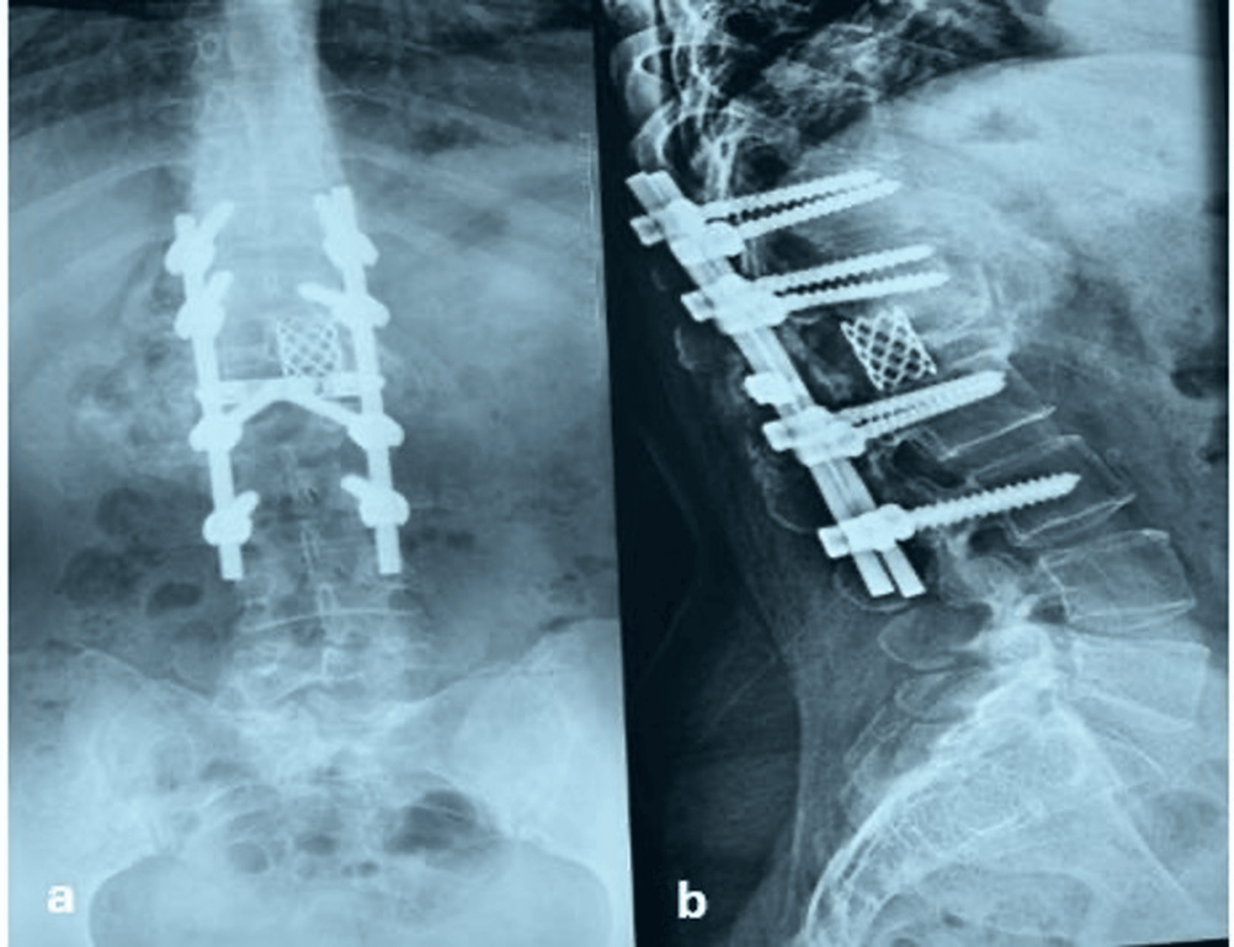
Post-surgical follow-up at nine months Postero-anterior and lateral thoracolumbar spine radiographs with proper placement of orthopedic implants are observed, with no evidence of loosening or adjacent vertebral destruction at the nine-month post-surgical follow-up.

Postoperatively, the patient recovered with stable vital signs. A neurological evaluation was performed at 24 hours, finding motor strength of four out of five from the L2-S1 motor root bilaterally, as well as sensation of two out of two from T11-S3 according to the Daniels scale, classifying it as an American Spinal Injury Association Impairment Scale (ASIA D) [12] incomplete spinal cord injury. Gait was also explored and was possible without the use of complementary aids. The wound remained intact, clean, and without signs of bleeding or dural injury. Discharge was indicated at 72 hours. Radiographs confirmed correction of kyphosis and stable instrumentation (Figures 3a, 3b). 

**Figure 4:**
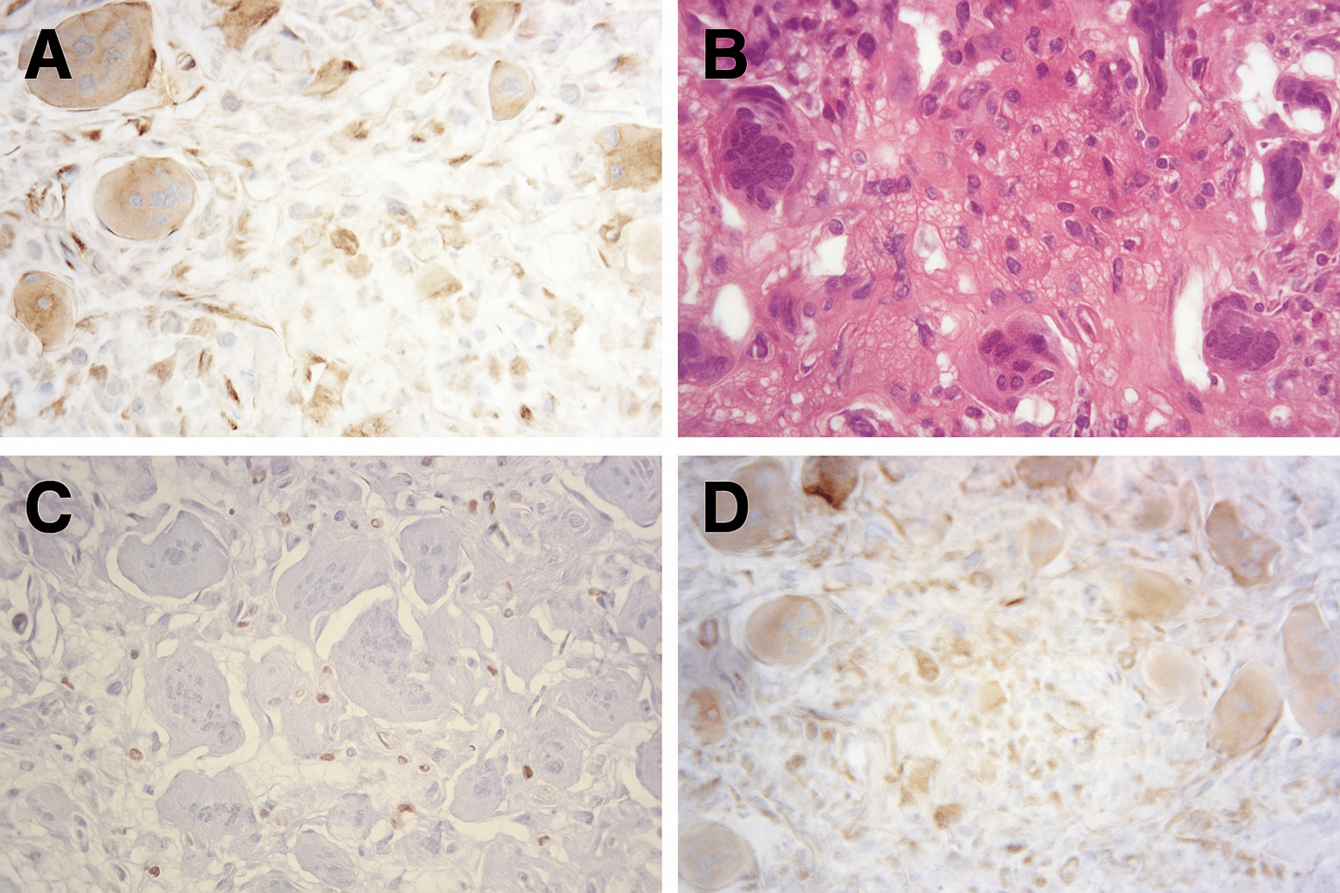
Histopathological and immunohistochemical findings of the L1 vertebral body lesion a) Immunohistochemical staining for special AT-rich sequence-binding protein 2 (SATB2), consistent with osteoblastic differentiation; b) Hematoxylin-eosin (H&E) staining showing multinucleated osteoclast-like giant cells interspersed with mononuclear stromal cells within a vascularized background; c) Immunohistochemical staining for P63 demonstrating nuclear positivity in stromal cells; d) Immunohistochemical staining for cyclin D1 revealing diffuse nuclear expression in stromal cells.

Final histopathology contradicted the initial biopsy. Ziehl-Neelsen staining was negative, excluding tuberculosis. Instead, sections revealed multinucleated osteoclast-like giant cells with mononuclear stromal proliferation. Immunohistochemistry showed positivity for cyclin D1, tumor protein 63 (P63), and special AT-rich sequence-binding protein 2 (SATB2), confirming the diagnosis of GCT (Figures 4a-4d).

She was discharged after 72 hours with referral to oncology for follow-up. Denosumab was started (120 mg subcutaneously on days one, eight, 15, and then every four weeks), along with calcium/vitamin D supplementation.

**Figure 5:**
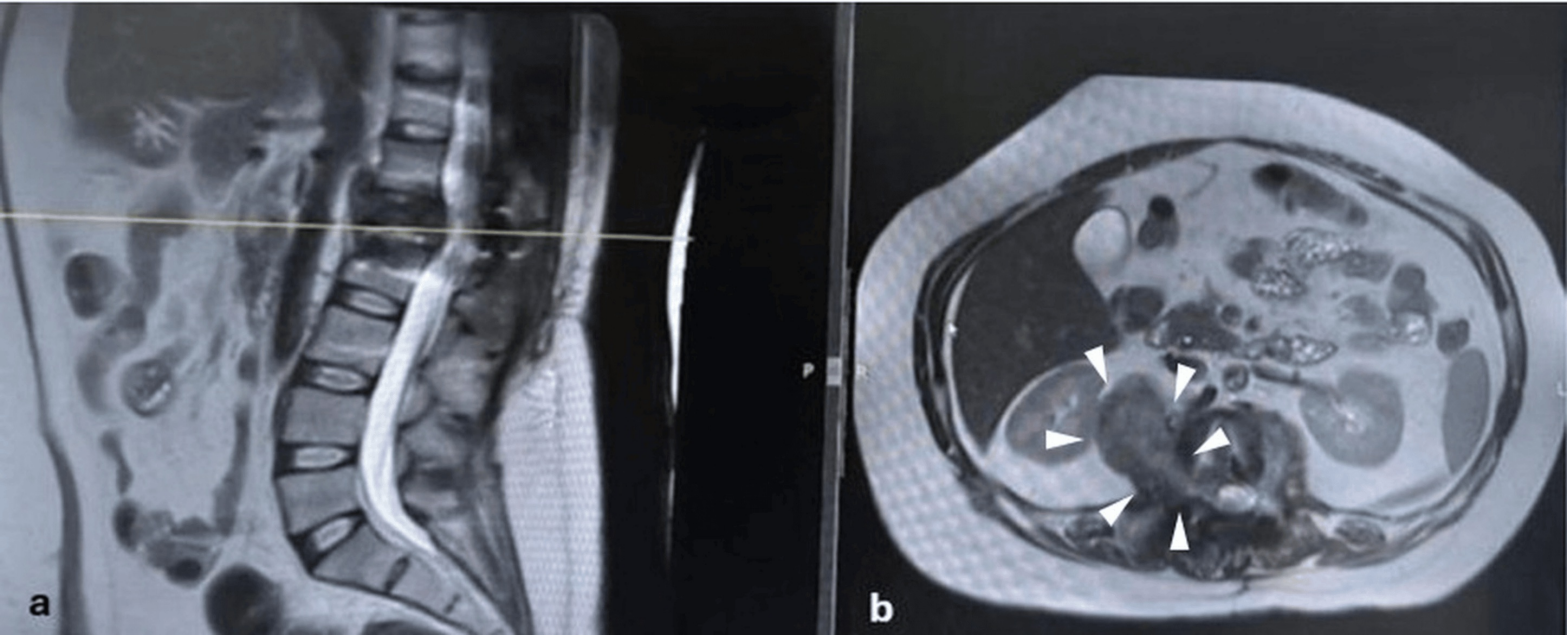
Sagittal and axial contrast-enhanced magnetic resonance imaging (MRI) of the lumbar spine at eight months follow-up a) Sagittal T2-weighted MRI demonstrating the lumbar spinal canal. The yellow reference line marks the level of the axial slice shown in panel b; b) Axial T2-weighted MRI at the corresponding level reveals a space-occupying lesion within the spinal canal (arrowheads), producing significant compression of the neural elements.

Eight months postoperatively (January 2025), contrast-enhanced MRI showed a lobulated T2-hypointense mass in the right medial perirenal space tracking along the psoas, between L1 and the right kidney, consistent with locoregional recurrence (Figures 5a, 5b).

The patient subsequently underwent laparotomy with retroperitoneal tumorectomy, yielding a 90 g heterogeneous mass; histopathology again confirmed GCT.

She began adjuvant radiotherapy for residual disease (planned 27 fractions, 54 Gy). At the time of reporting, she had completed 14 fractions with only mild fatigue/weakness, and follow-up imaging demonstrated ~60% tumor regression.

## Discussion

This case highlights a rare and diagnostically challenging presentation of a thoracolumbar spinal GCT that was initially misinterpreted as tuberculosis due to Ziehl-Neelsen-positive staining. The novelty of this report lies in the combination of false-positive Ziehl-Neelsen staining, the tumor’s thoracolumbar location, and the requirement for staged posterior instrumentation followed by retroperitoneal tumorectomy, a management sequence that is infrequently described in the literature.

The key diagnostic and therapeutic considerations are the following: GCTs typically present in patients between 20 and 40 years of age, show a slight female predominance, and most commonly arise in the sacrum [5,13-15]. Our patient, a 34-year-old woman with a thoracolumbar lesion, is consistent with this usual demographic profile but represents an atypical spinal location [5,13-15]. In endemic regions, GCTs may clinically and radiographically resemble infectious etiologies. When involving the vertebral body at the thoracolumbar junction, they pose significant technical challenges and carry a high risk of local recurrence despite aggressive multimodal therapy [14,16,17].

The most frequent presenting symptom is pain, reported in nearly all cases, while neurologic deficits occur in up to 72% of patients. In line with these reports, our patient developed progressive pain, kyphotic deformity, and transient paraplegia, though no palpable mass was detected. These findings highlight the importance of integrating clinical, radiographic, and histopathological features for accurate diagnosis [14,15].

Radiographically, spinal GCTs typically appear as expansile osteolytic lesions with a “soap bubble.” MRI often shows hypointense signals on T1- and T2-weighted sequences, particularly in aggressive tumors with extensive tissue invasion [5,14]. Our patient’s imaging showed these features, with vertebral collapse, spinal canal compromise, and aggressive local expansion. Differential diagnoses include metastatic lesions, chordoma, aneurysmal bone cyst, and spinal tuberculosis. Given her residence in a tuberculosis-endemic area, infection was strongly suspected initially, though histopathology ultimately confirmed GCT with osteoclast-like giant cells and stromal proliferation [5-9].

The management of spinal GCT is complex, particularly in the thoracolumbar region, where complete resection is difficult. According to a retrospective survival analysis of patients with Enneking stage III GCT [13], total en bloc spondylectomy (TES), when technically feasible and safe, is associated with significantly lower recurrence rates (around 20-25% or less in large series) compared to intralesional procedures. Compared to TES, recurrence after subtotal resection is 61.3%, and subtotal or intra-lesional resections in the spine carry higher risks of tumor contamination and local recurrence, especially in the epidural space [6,16,17].

The patient received multidisciplinary care, initially under the independent, concurrent involvement of the Spine Surgery and Infectious Disease services. Following confirmation of the diagnosis of GCT, Medical Oncology and Surgical Oncology were subsequently incorporated, with each specialty contributing to treatment planning without a formal tumor-board structure. Despite this coordinated involvement, early recurrence occurred within months, underscoring the aggressive behavior of incompletely controlled spinal GCT.

Adjuvant therapies play a pivotal role when total resection is not feasible. Preoperative selective arterial embolization can reduce tumor vascularity and intraoperative blood loss or serve as palliation in inoperable cases. For instance, radiotherapy has been employed for residual or unresectable tumors, but concerns remain about local recurrence and rare radiation-induced sarcomatous transformation into osteosarcoma, fibrosarcoma, or pleomorphic sarcoma. Follow-up with MRI (generally at 12-24 weeks of treatment) shows significant ossification of the lesion border and a reduction in T2 signal, which reflects a decrease in cellularity and edema [8,10,14].

Denosumab, a monoclonal antibody targeting receptor activator of nuclear factor kappa-B ligand (RANKL), has shown substantial tumor reduction, with reported regression rates up to 86% at six months. Generally, three to six (three to six doses) are needed to induce ossification without excessively increasing the risk of recurrence or malignancy. Studies report a median reduction in tumor size (volume) of approximately 5 to 6 mm after 24 weeks of therapy. The lytic lesion transforms into a sclerotic (hardened) lesion at the periphery, which is the desired radiological response [7,8,10,11,18].

In our case, denosumab was initiated immediately after the index surgery. Despite an initial radiographic response, locoregional recurrence was detected at eight months and was managed with retroperitoneal tumorectomy followed by adjuvant radiotherapy.

## Conclusions

Thoracolumbar GCT remains a rare and technically demanding condition because it arises in a region where small delays in diagnosis or treatment can result in neurologic decline and irreversible deformity. This case illustrates the diagnostic uncertainty that can arise when a GCT presents with clinical and histopathologic features that resemble infection, particularly in endemic areas. Even after an appropriately planned and executed surgical intervention with en bloc corpectomy, reconstruction, and long-segment stabilization, the tumor recurred early. This highlights the unpredictable behavior of spinal GCT and the need for close postoperative surveillance since recurrence can develop despite the correct application of contemporary treatment principles.

The patient benefited from coordinated evaluation by multiple specialties, including spine surgery, infectious disease, oncology, and surgical oncology, although the approach was not structured as a traditional tumor board. The recurrence in this context underscores that multidisciplinary care alone does not guarantee durable control in aggressive spinal GCT, especially when complete wide resection is limited by anatomic constraints. This case reinforces the importance of long-term follow-up and individualized management strategies, along with a high index of suspicion for recurrence even when the initial response is favorable.
